# Prepubertal Dietary and Plasma Phospholipid Fatty Acids Related to Puberty Timing: Longitudinal Cohort and Mendelian Randomization Analyses

**DOI:** 10.3390/nu13061868

**Published:** 2021-05-30

**Authors:** Tuck Seng Cheng, Felix R. Day, John R. B. Perry, Jian’an Luan, Claudia Langenberg, Nita G. Forouhi, Nicholas J. Wareham, Ken K. Ong

**Affiliations:** 1 MRC Epidemiology Unit, Institute of Metabolic Science, Cambridge Biomedical Campus Box 285, University of Cambridge School of Clinical Medicine, Cambridge CB2 0QQ, UK; tuckseng.cheng@mrc-epid.cam.ac.uk (T.S.C.); felix.day@mrc-epid.cam.ac.uk (F.R.D.); john.perry@mrc-epid.cam.ac.uk (J.R.B.P.); jianan.luan@mrc-epid.cam.ac.uk (J.L.); claudia.langenberg@mrc-epid.cam.ac.uk (C.L.); nita.forouhi@mrc-epid.cam.ac.uk (N.G.F.); nick.wareham@mrc-epid.cam.ac.uk (N.J.W.); 2 Department of Paediatrics, University of Cambridge, Cambridge CB2 0QQ, UK

**Keywords:** ALSPAC, fatty acids, puberty timing, prospective study, Mendelian Randomization

## Abstract

Dietary intakes of polyunsaturated, monounsaturated and saturated fatty acids (FAs) have been inconsistently associated with puberty timing. We examined longitudinal associations of prepubertal dietary and plasma phospholipid FAs with several puberty timing traits in boys and girls. In the Avon Longitudinal Study of Parents and Children, prepubertal fat intakes at 3–7.5 years and plasma phospholipid FAs at 7.5 years were measured. Timings of Tanner stage 2 genital or breast development and voice breaking or menarche from repeated reports at 8–17 years, and age at peak height velocity (PHV) from repeated height measurements at 5–20 years were estimated. In linear regression models with adjustment for maternal and infant characteristics, dietary substitution of polyunsaturated FAs for saturated FAs, and higher concentrations of dihomo-γ-linolenic acid (20:3n6) and palmitoleic acid (16:1n7) were associated with earlier timing of puberty traits in girls (*n* = 3872) but not boys (*n* = 3654). In Mendelian Randomization models, higher genetically predicted circulating dihomo-γ-linolenic acid was associated with earlier menarche in girls. Based on repeated dietary intake data, objectively measured FAs and genetic causal inference, these findings suggest that dietary and endogenous metabolic pathways that increase plasma dihomo-γ-linolenic acid, an intermediate metabolite of n-6 polyunsaturated FAs, may promote earlier puberty timing in girls.

## 1. Introduction

Global secular trends towards earlier timing of puberty [1,2] represent an increasingly important public health issue. Substantial evidence has demonstrated that an earlier timing of puberty is associated with poorer mental and physical health in later life [3,4,5,6,7]. While the underlying mechanisms remain unclear, greater childhood growth and obesity have been associated with both earlier puberty [8] and adverse health outcomes [9,10,11,12,13]. These findings suggest that the associations between puberty timing and subsequent health may be modifiable by dietary intake and adiposity. In addition to reported associations with higher total energy intake and higher protein intakes on earlier menarche in girls [14,15], fat quality, indicated by different types of fatty acids (FAs)–saturated (SFA), monounsaturated (MUFA) or polyunsaturated (PUFA), is a key dietary determinant of cardiometabolic health [16] and may potentially influence puberty timing [15]. Furthermore, due to their highly distinct biochemical properties, individual FAs, either from dietary intakes or endogenous synthesis, can have different physiological and metabolic effects [17], probably including the progression of pubertal development [15].

Few prospective studies have reported inconsistent associations of dietary fat quality with puberty timing, which was typically assessed as age at menarche among girls [18]. Prospective studies have consistently reported no relationship between SFA intake and puberty timing in girls [19,20,21], consistent with the results of most trials of interventions that reduced SFA intakes in boys and girls [22,23,24,25], although in one trial, boys in the intervention arm had earlier genital development than controls [25]. Some studies found that higher PUFA intake [19], specifically n-3 PUFA [26], was associated with earlier puberty timing, while others did not observe any association [20,21]. A study further estimated two individual dietary FAs and reported that lower oleic acid (18:1n9) but not linoleic acid (18:2n6) was associated with earlier menarche [26]. These findings, however, are in contrast to the general notion that PUFA is considered a healthier alternative to SFA [17]. Furthermore, previous studies mostly relied on a single parent- or self-report measure of dietary intake at a particular time-point and puberty timing in girls, and did not consider isocaloric macronutrient substitutions in analyses. To our knowledge, no study has examined objective biomarkers of FAs, which is more robust to measurement error and allows assessment of individual FAs and the metabolic pathways, in relation to puberty timing.

The present study aimed to investigate the longitudinal associations between fat quality during prepubertal childhood and puberty timing traits in boys and girls. Firstly, we aimed to confirm the previous association between higher PUFA intake and earlier menarche in girls [19] in the same cohort (the Avon Longitudinal Study of Parents and Children (ALSPAC) [27,28]), but with more comprehensive puberty data and an integrative analytical approach to combine the childhood dietary data. Secondly, we further explored the associations using objectively measured plasma phospholipid FA concentrations, specifically: n-3 and n-6 PUFAs and their metabolites. Thirdly, we examined the associations for the two most abundant monounsaturated FAs (MUFAs) in adipose tissue (i.e., palmitoleic acid (16:1n7) and oleic acid (18:1n9)) [29,30], given the well-established relation between adiposity and puberty timing [8]. Finally, we examined the likely causal nature of any observed associations between specific FA concentrations and puberty timing, using Mendelian Randomization (MR) analyses, which take the advantage of the natural randomized allocation of allelic variation in genes affecting specific exposures [31,32].

## 2. Materials and Methods

### 2.1. Study Population

ALSPAC is an ongoing population-based birth cohort study that initially recruited 14,541 pregnant mothers, who delivered between 1 April 1991 and 31 December 1992, from the former Avon county of southwest England, United Kingdom [27,28]. The cohort consisted of 14,901 children, of whom 13,988 children were those alive at age 1 year from enrolled pregnancies; and 913 additional children were recruited from age 7 years onwards. Children were followed-up at regular intervals using questionnaire and clinic-based assessments, and non-fasting venous blood samples were taken at 7.5 years. Further information about the study including details of all the data available are at: http://www.bristol.ac.uk/alspac/researchers/our-data/ (accessed on 1 April 2021). Informed consent was obtained from participants following the recommendations of the ALSPAC Ethics and Law Committee at the time, and biological samples were collected in accordance with the Human Tissue Act (2004). Ethical approval for this cohort study was obtained from the ALSPAC Ethics and Law Committee and the Local Research Ethics Committees (E1808 Children of the Nineties; 49/89 Children of Nineties; 90/8 Children of the Nineties); more details at: (http://www.bristol.ac.uk/alspac/researchers/research-ethics/) (accessed on 1 April 2021).

### 2.2. Dietary Fat Quality

Food frequency questionnaires at ages ~3 (mean ± standard deviation (SD): 3.22 ± 0.10 years), ~4 (4.54 ± 0.10 years), and ~7 years (6.79 ± 0.11 years) and 3-day food diary at age ~7.5 years (7.5 ± 0.17 years) were administered to the main carers to assess their child’s dietary intakes. Further information including the plausibility of reporting can be found elsewhere [33,34] and in Appendix A.

In summary, the food frequency questionnaire was adapted at each age to cover age-appropriate child’s diets and validated against 3-day food diaries administered to a 10% subcohort [19]. The assessed items were the current frequency of intakes of ~60 food/drink groups, each with five response options ranging from ‘*never or rarely*’ to ‘*more than once a day*’ [33,34]. Five food/drink groups, normally consumed daily in a variety of types (bread, spreading and frying fat, milk, tea and coffee), were further covered in more detail. Nutrient intakes (per day) were estimated by multiplying the reported frequency of each food consumed by the nutrient content (including PUFA, MUFA, and SFA) of a standard portion tailored to the age of the child (from the 5th edition of McCance and Widdowson’s The Composition of Foods and its supplements [35]) [33,34]. Further, dietary n-3 PUFA, eicosapentaenoic acid (20:5n3), and docosahexaenoic acid (22:6n3) were estimated from the reported intakes and fatty acids composition of a list of fish groups (i.e., shellfish, white fish in breadcrumbs or batter, white fish without coating, tuna, and other fish), since seafood was the predominant source of essential n-3 PUFA in the diet within this population.

The 3-day food diaries were posted to participants prior to attending research clinics to record all foods and drinks consumed by the child in household measures over 3 days (consecutive or non-consecutive, comprising one weekend day and two weekdays). Energy and nutrient contents (including PUFA, MUFA, and SFA) of each food consumed was estimated using DIDO software [36], based on a database comprising the 5th edition of McCance and Widdowson’s Food Composition tables, as well as information from manufacturers and the National Diet and Nutrition Survey [33,34].

Dietary intake values between ages 3 to 7.5 years were summarized using random intercepts linear regression models for total energy intake and each macronutrient (PUFA, MUFA, SFA, carbohydrate, and protein) [37]. In each model, age was centered to 6 years old and the individual intake of each macronutrient at age 6 years was estimated by summing the model constant value (i.e., the population mean intake at age 6 years) and the Best Unbiased Linear Predictor estimate (i.e., the difference between the person-specific value and the population mean). The predicted dietary fat quality intake was moderately to highly correlated with reported intakes at 3–7.5 years (Pearson’s correlation coefficients (r) = 0.64 to 0.78). Similar prediction was performed for dietary n-3 PUFA, and for eicosapentaenoic acid (20:5n3) and docosahexaenoic acid (22:6n3) from fish sources using intakes between ages 3 to 7 years for supplementary analyses (Pearson’s correlation coefficients = 0.71–0.85).

### 2.3. Plasma Phospholipid FA Concentrations

Plasma FAs stored at −70 °C were profiled at the National Institute on Alcohol Abuse and Alcoholism, Rockville, Maryland, United States of America (USA) in 2009–2010 [38]. Briefly, the assay methods included: (i) transmethylation of lipids with acetyl chloride and methanol using a simplified method based on the Lepage and Roy procedure [39] by a high throughput automated method [40], (ii) internal calibration by adding internal standard to each assay, followed by a second standard to quantify the exact amount of internal standard in every batch for ongoing assay of experimental variability, (iii) transmethylation and extraction of FAs using Freedom Evo Instrument 200 (TECAN Trading AG, Switzerland) and (iv) separation of FAs using gas chromatography 6890 Plus LAN system (Agilent Technologies, Inc, Santa Clara, California, CA, USA). The within and between day imprecision was 3.26 ±1.2% and 2.95 ± 1.6% for FA concentrations ranging 1–600 μg/mL plasma. A total of 23 FAs were identified, which comprised 12 PUFAs (including 4 long-chain n-3 PUFAs, 7 n-6 PUFAs), 5 MUFAs (including palmitoleic acid (16:1n7) and oleic acid (18:1n9)), and 6 SFAs (3 short-even-chain SFAs, 3 long-even-chain SFAs). Plasma phospholipid FA concentrations were expressed as percentage of total phospholipid FAs (μg%) and were age-adjusted by obtaining the residuals from linear regression models. Several product-to-precursor ratios of FAs were further calculated to estimate a) biological processes: (i) activity of Δ6 (γ-linolenic acid (18:3n6)/linoleic acid (18:2n6)) and Δ5 (arachidonic acid (20:4n6)/dihomo-γ-linolenic acid (20:3n6)) desaturase enzymes, (ii) the conversion of linoleic acid to dihomo-γ-linolenic acid (20:3n6/18:2n6), and (iii) stearoyl-CoA desaturase-1 (palmitoleic acid (16:1n7)/palmitic acid (16:0) and oleic acid (18:1n9)/stearic acid (18:0); and b) the balance between: (i) two major PUFA subclasses (total n-6 PUFAs/total n-3 PUFAs) and (ii) MUFAs and SFAs (total MUFAs/total SFAs).

### 2.4. Puberty Timing Traits

Children’s pubertal development was reported annually by caregivers (mainly parents) from ages 8 to 13 years and by children themselves from ages 14 to 17 years, using an adapted version of the Pubertal Development Scale [41]. Genital development in boys and breast development in girls were reported from a choice of five line-drawn images of Tanner stages ranging from prepuberty (stage 1) to postpuberty (stage 5). Voice breaking status in boys was reported as: ‘no’, ‘partial’, or ‘total’. Menarche in girls was recorded as the date (month and year) and/or age (in years) at first occurrence of menstruation.

Repeated measures of pubertal development were combined to produce individual estimates of: (1) age at pubertal onset, based on Tanner stage 2 genital (G2) or breast (B2) development in boys and girls, respectively, and (2) age at pubertal completion, based on voice breaking or menarche, respectively. Ages at G2, B2, and voice breaking were calculated as the midpoint age between the first pubertal appearance and the previous annual report stating prepubertal status. Age at menarche was considered as the earliest reported age (from reported date or age). Details on the estimations of puberty timing can be found in Appendix B.

Age at peak height velocity (PHV), an objective measure of puberty timing, was derived from 61,290 measurements of standing height between ages 5–20 years, separately in boys (*n* = 5137) and girls (*n* = 5099), by transformation of the random age intercept that indicates the timing of the height growth spurt using SuperImposition by Translation and Rotation analysis [42].

### 2.5. Phenotypic Analyses

Inclusion criteria for this analysis were: white ethnicity (reported by mother and her partner), gestational age ≥36 weeks, singleton pregnancy and mother’s age at delivery ≥18 years. Among 10,789 ALSPAC children who met these criteria, 7526 children had reported puberty timing and 3263 did not (Figure S1); these groups were compared using chi-squared tests for categorical variables and *t*-tests for continuous variables.

To test the associations between dietary fat quality and puberty timing, multivariable linear regression models were performed separately in boys and girls, with adjustment for maternal characteristics (age at delivery, passive and active smoking during pregnancy, age at menarche, education, pre-pregnancy body mass index, parity), highest maternal or mothers’ partner’s occupational socioeconomic group at 18 weeks of gestation, and infant characteristics (birth weight, gestational age, breastfeeding duration). We modelled the substitution of PUFA for SFA in an isocaloric diet using the *nutrient density method* which includes percentages of energy contributed by PUFA, MUFA, carbohydrate and protein, and total energy intake [43]. The resulting beta value for PUFA is interpreted as the effect of each 5% increase in PUFA as SFA (the only remaining macronutrient) decreases by 5%, while other macronutrients and total energy intakes are held constant. The substitution model was re-tested using the *residual method* which includes the residuals from the regression of each macronutrient intake on total energy intake and thus accounts for potential underreporting of dietary intakes [44]. The substitution of MUFA for SFA was examined similarly using the *nutrient density and residual methods* as supplementary analyses.

The associations of total and individual plasma concentrations of n-6 PUFAs and MUFAs (i.e., palmitoleic acid and oleic acid) with puberty timing were examined using multivariable linear regression models, adjusted for maternal and infant characteristics, and total energy intake at 6 years (summarized from habitual energy intakes at 3–7.5 years). A series of sensitivity analyses were also conducted as follows: a) further adjustment for intakes in grams at 6 years (from intakes at 3–7.5 years) of foods that are unlikely major sources of PUFAs (total intakes of red meat, chicken, fruits and vegetables, dairy and eggs, and sugar confectionery), and b) additionally adjusted for possible dietary sources of PUFAs (total intakes of fish, and cereals and nuts) [45]. The basic model also further adjusted for body mass index at 7.5 years to take into account potential confounding and/or mediating effects of adiposity. We also compared findings from basic models for self-reported dietary and objectively measured n-3 PUFAs. Other supplementary analyses included the basic model for remaining MUFAs (including vaccenic acid (18:1n7) which is a precursor of trans-palmitoleic acid to explore whether the effect of palmitoleic acid (16:1n7) was due to cis-palmitoleic acid or trans-palmitoleic acid, erucic acid (20:1n9), nervonic acid (24:1n9)), two SFA categories, and the FA ratios described above.

We used multiple imputation by chained equations with 50 imputed datasets [46] for all analyses, to account for missing data on dietary intake (missing *n* = 183 to 317, 2.4 to 4.2%), plasma phospolipid FAs (missing *n* = 3425, 45.5%), and covariates (missing *n* = 16–1205, 0.2 to 16.0%; or missing at least one covariate *n* = 2484, 33%) under the assumption of missing at random.

Statistical analyses were performed using Stata 15.1 (StataCorp. 2017. Stata Statistical Software: Release 15. College Station, TX, USA: StataCorp LLC).

### 2.6. Mendelian Randomization Analyses

We conducted two-sample MR analyses to estimate the likely causal nature of identified plasma FAs to puberty timing associations. To identify genetic instruments, we explored genome-wide association study (GWAS) data on plasma FA concentrations in up to 27,286 adults from the European Prospective Investigation into Cancer and Nutrition (EPIC)–InterAct cohort [47]. More details on the EPIC-InterAct can be found in Appendix C and elsewhere [47]. FA levels were expressed as percentage of total phospholipid FAs (mol%). Analyses were conducted by generalized linear mixed models adjusted for age, sex, and population structure using BOLT-LMM [48] and genome-wide complex trait analysiswas used to identify independent signals. Genetic instruments were selected as those variants with minor allele frequency ≥1%, *p* value < 5.0 × 10^−8^ for association with the target FA, and weaker or null associations with concentrations of other FAs as well as of high- and low-density lipoproteins in the UK Biobank [49,50]. Associations between the identified genetic instruments and puberty timing were tested using published GWAS summary statistics for age at menarche on up to 329,345 women [51].

Inverse variance-weighted (IVW) MR analyses were computed using the “TwoSampleMR” package [52] in R software. Where there were multiple genetic variants identified for each FA, sensitivity models were computed to address possible pleiotropy and heterogeneity between variants (MR-Egger [53], weighted median MR, and penalized weighted median MR [54]).

## 3. Results

### 3.1. Children’s Characteristics

This study analyzed data on 7526 children (3654 boys and 3872 girls). Compared to children with missing puberty timing (*n* = 3263), mothers of the children included in the final sample were more likely to be older, nulliparous, from higher socioeconomic groups, have higher education level, and less likely to smoke or have second-hand smoke exposure during pregnancy (Table S1). Included children were more likely to be girls, were breastfed for longer, and tended to have modestly lower intakes of total energy and macronutrients at age 6 years than children with missing data on puberty timing.

Table 1 summarizes childhood macronutrient intakes, plasma phospholipid FAs at age 7.5 years, and puberty timing traits in boys and girls. Compared to boys, girls consumed lower total energy, relatively less energy from carbohydrate, marginally more energy from protein and dietary MUFA. In plasma samples, girls versus boys had modestly higher total FAs and concentrations of α-linolenic acid, docosahexaenoic acid, palmitoleic acid and oleic acid, and modestly lower concentrations of total and several individual n-6 PUFAs and docosapentaenoic acid.

Table S2 shows the correlations between dietary and plasma phospholipid PUFAs. In both boys and girls, total dietary PUFA intake was weakly correlated with concentrations of total n-6 PUFAs, linoleic acid, and eicosadienoic acid (*r* = 0.11 to 0.22). Dietary n-3 PUFA was modestly correlated with concentrations of total n-3 PUFAs and docosahexaenoic acid (*r* = 0.22 to 0.32). Additionally, there were weak correlations between dietary intakes and plasma concentrations of the specific n-3 PUFAs: eicosapentaenoic acid (*r* = 0.13 and 0.12, in boys and girls, respectively) and docosahexaenoic acid (*r* = 0.28 and 0.32).

Table S3 shows the correlations between plasma phospholipid PUFAs and major MUFAs. Total n-6 PUFAs was strongly positively correlated with linoleic acid (*r* = 0.92), and moderately negatively correlated with total and major MUFAs (*r* = −0.60 to −0.68). Total n-3 PUFAs was strongly positively correlated with eicosapentaenoic acid (*r* = 0.76) and docosahexaenoic acid (*r* = 0.86). Total MUFAs was very strongly positively correlated with oleic acid (*r* = 0.99).

### 3.2. Dietary Fat and Puberty Timing

Table 2 shows the association between childhood PUFA intake and puberty timing in boys and girls. In nutrient density models, the substitution of dietary PUFA for SFA was associated in girls with earlier B2 (β = −0.78 years per 5% higher PUFA intake, 95% confidence interval (CI): −1.31, −0.25) and PHV (β = −0.40, 95% CI: −0.72, −0.09), and a tendency to earlier menarche (β = −0.32, 95% CI: −0.71, 0.07). In contrast, no association was seen in boys. These findings remained robust in residual models. No association between MUFA intake and puberty timing in boys or girls was found (Table S4).

### 3.3. Plasma Phospholipid FAs and Puberty Timing

Table 3 shows the associations of plasma n-6 PUFAs and major MUFAs with puberty timing in boys and girls. Higher concentrations of dihomo-γ-linolenic acid and palmitoleic acid were associated in girls with earlier B2 (*β* = −0.09 years per SD, 95% CI: −0.16, −0.02; and *β* = −0.13, 95% CI: −0.20, −0.06, respectively), PHV (*β* = −0.07, 95% CI: −0.11, −0.03; and *β* = −0.06, 95% CI: −0.10, −0.02), and menarche (*β* = −0.06, 95% CI: −0.11, −0.01; and *β* = −0.06, 95% CI: −0.11, −0.01), but not with any puberty timing trait in boys. These associations persisted after further adjustment for non-major food sources and food sources of PUFAs at 6 years (Table S5). However, the associations with palmitoleic acid were completely attenuated with further adjustment for body mass index at 7.5 years (Table S6).

There was no strong evidence for any association of dietary intake or plasma concentration of n-3 PUFAs with puberty timing in boys or girls (Table S7).

Table S8 shows the associations of other MUFAs, SFA categories, and ratios of FAs at 7.5 years with puberty timing. Higher ratios of 20:3n6 to 18:2n6 (indicative of higher conversion of linoleic acid to dihomo-γ-linolenic acid activity) and 16:1n7 to 16:0 (indicative of higher Stearoyl-CoA desaturase-1 activity) were associated with earlier timing of puberty traits in girls but not in boys. No association of vaccenic acid, erucic acid, nervonic acid, SFA categories, and other ratios of FAs with puberty timing was found.

### 3.4. Genetically Inferred Effects on Age at Menarche

To confirm the observed phenotypic associations with earlier puberty timing in girls, we selected one variant very strongly associated with plasma dihomo-γ-linolenic acid concentrations (rs12928099 in *PDXCD1*, encoding pyridoxal dependent decarboxylase domain containing 1: *p* = 2.3 × 10^−196^) as an instrument for genetic causal modelling. This variant conferred *higher* concentration of that metabolite and had weaker effects on *lower* concentrations of its immediate upstream (γ-linolenic acid) and downstream (arachidonic acid) metabolites, suggesting a primary effect on *higher* dihomo-γ-linolenic acid concentrations (Table S9 and Figure S2). We also selected one variant strongly and specifically associated with palmitoleic acid concentrations (rs603424 near *SCD1*, encoding Stearoyl-CoA desaturase-1: *p* = 6.2 × 10^−38^).

Genetically predicted higher plasma dihomo-γ-linolenic acid was associated with earlier menarche in girls (*β* = −0.05 years per SD; 95% CI: −0.09 to −0.01), and the genetic effect estimate was very similar to the observed phenotypic association (*β* = −0.06; 95% CI: −0.11, −0.01). Sensitivity models, including two additional variants with weaker associations with dihomo-γ-linolenic acid (rs721399 and rs499974) (Table S9), showed similar genetic associations between dihomo-γ-linolenic acid and earlier menarche in IVW (*β* = −0.04; −0.09 to 0.02), weighted median (*β* = −0.05; −0.09 to −0.01) and penalized weighted median models (*β* = −0.04; −0.09 to −0.01) (Table S10). There was no evidence of horizontal pleiotropy (MR-Egger intercept = 0.01, *p* = 0.6).

Genetically predicted plasma palmitoleic acid was not associated with age at menarche (*β* = 0.08; 95% CI: −0.01, 0.16), and the genetic effect estimate did not overlap the observed phenotypic association (*β* = −0.06 (−0.11, −0.01). Sensitivity models, including an additional variant with weaker association with palmitoleic acid (rs4962238), also showed no association with age at menarche (Table S10).

## 4. Discussion

In this prospective British birth cohort study, higher habitual intake of PUFA substituting for SFA, and higher plasma concentrations of an intermediate metabolite of n-6 PUFAs (dihomo-γ-linolenic acid) during prepubertal childhood, were associated with subsequent earlier timing of several puberty traits, in girls but not boys. Consistently, MR analyses supported a likely causal effect of higher dihomo-γ-linolenic acid concentrations on earlier menarche in girls. Associations between higher plasma phospholipid concentrations of palmitoleic acid, one of the most abundant MUFAs in adipose tissue, and earlier puberty timing in girls were also seen, but this association was not confirmed in MR analyses.

Our study significantly extends previous analyses of dietary fat quality and puberty timing [19,20,21,22,23,24,25,26]. Our findings are consistent with the previous ALSPAC (*n* = 3298) [19] report which described associations of higher PUFA intakes at 3 and 7 years (separately) with higher odds of early menarche (before age 12 years and 8 months). Here, we advance those findings in many ways: by including data on both boys and girls, by combining the repeated dietary assessments in childhood, by utilising longitudinal data on several puberty traits from early adolescence to young adulthood, and by including objective measures of FAs (plasma phospholipid FA concentrations) and puberty timing (PHV), as well as analyses of isocaloric macronutrient substitution and genetic causal modelling. Furthermore, our null findings of modelled dietary substitution of MUFA for SFA corroborate those from the 3-year follow-up trials (Dietary Intervention Study in Children) that promoted lower intakes of SFA (<8% of total energy intake) and PUFA (<9%) and higher intake of MUFA (>11% within 28% energy contributed by total fat intake) [22,23], and the randomized prospective Special Turku Coronary Risk Factor Intervention Project that provided dietary counselling to maintain a ratio of 1:2 for SFA and MUFA or PUFA intakes since 7 months old [24,25]. Those previous trials reported similar timings of genital development in boys [23,24] and breast development and age at menarche in girls [22,24,25] between intervention and control groups. Unlike our findings using individual plasma phospholipid FAs at 7.5 years (before puberty onset), one study in the United States (*n* = 194) estimated oleic acid and linoleic acid by FFQ at age 10 years, and found an association between lower dietary oleic acid and early menarche [26], which was not seen in the present study.

We found strong coherence between findings using self-reported dietary fat intake, plasma phospholipid FA concentrations, and genetic causal modelling. Higher dietary intake of PUFA and higher plasma concentration of the intermediate metabolite of n-6 PUFAs (i.e., dihomo-γ-linolenic acid) were consistently associated with earlier puberty timing in girls; these phenotypic associations were consistently null in boys. Additionally, dietary n-3 PUFAs (eicosapentaenoic acid and docosahexaenoic acid), and their respective biomarkers, consistently showed no association with puberty timing. It is well-recognized that Western diets typically contain much more n-6 than n-3 PUFAs [55], which cannot be synthesized endogenously [17]. While linoleic acid is the greatly ubiquitous n-6 PUFA [56], dihomo-γ-linolenic acid exists in a small amount in animal sources such as meat and eggs [57]. After consumption, linoleic acid is catalyzed to γ-linolenic acid which is rapidly elongated to dihomo-γ-linolenic acid, most of which accumulates—some dihomo-γ-linolenic acid is further converted to arachidonic acid but this diminishes with increasing availability of dihomo-γ-linolenic acid [58]. Although oxidative metabolites of dihomo-γ-linolenic acid have anti-inflammatory effects [58], dihomo-γ-linolenic acid concentrations are positively associated with risks for type 2 diabetes and insulin resistance [57,59,60], which may in turn potentially increase bioavailability of female sex steroids particularly, and hence promote earlier pubertal development [8] in girls.

Dietary MUFA intake and plasma phospholipid concentrations of major MUFAs (i.e., palmitoleic acid and oleic acid) are not directly comparable, since MUFAs can be sourced from the diet or endogenously synthesized from SFAs. Palmitoleic acid is also low in dietary concentration and rapidly oxidized [29]. This might explain why we observed no association for MUFA intake with earlier puberty timing but negative associations for plasma palmitoleic acid with puberty timing in girls, which largely attenuated on additional adjustment for body mass index, and also no overlap between phenotypic and MR effect estimates. Hence, the observed association of plasma palmitoleic acid with earlier puberty timing may be partly driven by lower brown adipose tissue [61] or higher white adipose tissue which is predominantly composed of oleic acid (a precursor of palmitoleic acid) and palmitoleic acid [29,30].

We acknowledge several limitations in this study. Modest differences in some maternal and infant characteristics were found between children with and without data on puberty timing, however these factors were adjusted for in all analyses. We were unable to identify individuals with implausible reported energy intakes because body weight was not measured at 3–7 years. However, we addressed this issue by *residual method* substitution models which account for potential under-reporting of dietary intakes [62]. Furthermore, we combined dietary data from several timepoints to reduce random error and avoid multiple testing [37]. Dietary intakes were parent-reported and single timepoint plasma samples were analyzed after storage. Nonetheless, we observed the expected modest positive correlations between habitual childhood dietary fat quality intakes and their respective biomarkers. The data on other plasma phospholipid FAs such as odd-chain SFAs (pentadecanoic acid (15:0) and heptadecanoic acid (17:0)) and trans FAs were not available. We were unable to estimate several puberty timing traits in 20–30% of children due to uncorrectable inconsistencies in their repeated parent and/or self-reports, however we found consistent findings with PHV, an objective measure of puberty timing. Our observed phenotypic associations may be affected by residual confounding due to unmeasured factors such as physical activity (which was not measured in ALSPAC before age 7 years), however for dihomo-γ-linolenic acid we report similar findings using MR which is considered to be less influenced by such confounding. Inferences from MR analyses are subject to a number of assumptions. We aimed to avoid pleiotropy by selecting a single variant with a very strong and specific association with the target FA. Our genetic instrument for higher dihomo-γ-linolenic acid concentrations appeared to be specific for this FA. Our primary genetic instrument for palmitoleic acid is located near the gene encoding Stearoyl-CoA desaturase-1 which is the enzyme that catalyzes its biosynthesis. Finally, our phenotypic and MR analyses were conducted among white populations in the UK, which may limit the generalizability of our findings to other ethnicities and/or populations.

## 5. Conclusions

Our phenotypic analyses of repeated dietary intakes and objectively measured plasma FAs showed that higher habitual dietary intake of PUFAs, specifically higher concentrations of an intermediate metabolite of n-6 PUFAs (dihomo-γ-linolenic acid), were associated with earlier puberty timing in girls, but not in boys. Results of MR analyses further supported that increasing circulating plasma dihomo-γ-linolenic acid concentrations from dietary and endogenous metabolic pathways may promote earlier age at menarche in girls. Our findings suggest that there may be sex-specific individual fatty acid sensing pathway underlying pubertal development.

## Figures and Tables

**Table 1:** Macronutrient intakes, plasma phospholipid fatty acids, and puberty timing in boys and girls in the ALSPAC study.

	Boys (*n* = 3811)	Girls (*n* = 3919)	*p*
	Mean ± SD	Mean ± SD	
Dietary intakes predicted at age 6 years			
Total energy intake, kcal	1654 ± 159	1578 ± 148	<0.001
Total carbohydrate intake, g	207.2 ± 20.7	196.9 ± 19.5	<0.001
Total protein intake, g	56.6 ± 5.8	54.7 ±5.3	<0.001
Total fat intake, g	67.4 ± 7.4	64.4 ± 6.8	<0.001
Polyunsaturated fat intake, g	11.0 ± 1.3	10.6 ± 1.1	<0.001
Monounsaturated fat intake, g	21.8 ± 2.4	20.9 ± 2.2	<0.001
Saturated fat intake, g	27.3 ± 4.0	26.1 ± 3.6	<0.001
Percentage of energy from carbohydrate, %	50.1 ± 1.7	49.9 ± 1.6	<0.001
Percentage of energy from protein, %	13.8 ± 1.0	13.9 ± 0.9	<0.001
Percentage of energy from fat, %	36.5 ± 1.6	36.6 ± 1.4	0.049
Percentage of energy from polyunsaturated fat, %	6.0 ± 0.6	6.0 ± 0.5	0.419
Percentage of energy from monounsaturated fat, %	11.8 ± 0.6	11.8 ± 0.5	0.005
Percentage of energy from saturated fat, %	14.8 ± 1.5	14.8 ± 1.3	0.431
			
Plasma phospholipid fatty acids at 7.5 years			
Total fatty acids, μg/ml	2279.0 ± 521.8	2378.5 ± 547.4	<0.001
n-6 polyunsaturated fatty acids, μg%			
Total	39.9 ± 4.0	39.7 ± 3.9	0.034
Linoleic acid (18:2n6)	30.6 ± 3.2	30.6 ± 3.1	0.833
γ-Linolenic acid (18:3n6)	0.39 ± 0.14	0.37 ± 0.12	<0.001
Eicosadienoic acid (20:2n6)	0.21 ± 0.04	0.20 ± 0.04	0.029
Dihomo-γ-linolenic acid (20:3n6)	1.76 ± 0.37	1.70 ± 0.35	<0.001
Arachidonic acid (20:4n6)	6.5 ± 1.3	6.3 ± 1.3	0.001
Docosatetraenoic acid (22:4n6)	0.25 ± 0.05	0.24 ± 0.05	<0.001
Docosapentaenoic acid (22:5n6)	0.19 ± 0.05	0.18 ± 0.05	<0.001
n-3 polyunsaturated fatty acids, μg%			
Total	3.85 ± 0.80	3.87 ± 0.77	0.406
α-Linolenic acid (18:3n3)	0.71 ± 0.27	0.73 ± 0.29	0.026
Eicosapentaenoic acid (20:5n3)	0.64 ± 0.22	0.63 ± 0.20	0.086
Docosapentaenoic acid (22:5n3)	0.64 ± 0.14	0.61 ± 0.13	<0.001
Docosahexaenoic acid (22:6n3)	1.86 ± 0.52	1.90 ± 0.52	0.016
Monounsaturated fatty acids, μg%			
Palmitoleic acid (16:1n7)	1.27 ± 0.40	1.32 ± 0.41	<0.001
Oleic acid (18:1n9)	22.2 ± 3.2	22.6 ± 3.0	<0.001
			
Puberty timing, years			
Age at pubertal onset (breast or genital development)	8.7 ± 1.6	10.0 ± 1.6	<0.001
Age at peak height velocity	13.6 ± 0.9	11.7 ± 0.8	<0.001
Age at pubertal completion (voice breaking or menarche)	13.6 ± 1.9	12.7 ± 1.2	<0.001

**Table 2:** Substitution of dietary polyunsaturated fat for saturated fat intake predicted at 6 years old and puberty timing in the ALSPAC study.

Macronutrients	Age at Genital/Breast Development		Age at Peak Height Velocity		Age at Voice Breaking/Menarche	
	Adjusted β (95% CI)	*p*	Adjusted β (95% CI)	*p*	Adjusted β (95% CI)	*p*
Boys	*n* = 2619		*n* = 2215		*n* = 3017	
Nutrient density model (per 5% increase) ^1^	0.05 (−0.48, 0.59)	0.843	−0.12 (−0.44, 0.19)	0.446	−0.35 (−0.93, 0.22)	0.225
Residual model (per 10g increase) ^2^	0.17 (−0.48, 0.81)	0.612	−0.13 (−0.51, 0.24)	0.485	−0.48 (−1.17, 0.20)	0.168
						
Girls	*n* = 3204		*n* = 2509		*n* = 3414	
Nutrient density model (per 5% increase) ^1^	−0.78 (−1.31, −0.25)	0.004	−0.40 (−0.72, −0.09)	0.011	−0.32 (−0.71, 0.07)	0.103
Residual model (per 10 g increase) ^2^	−0.86 (−1.50, −0.22)	0.008	−0.45 (−0.83, −0.08)	0.018	−0.35 (−0.81, 0.11)	0.139

^1^ adjusted for maternal characteristics (i.e., age at delivery, passive and active smoking during pregnancy, age at menarche, education, pre-pregnancy body mass index, parity), household highest socioeconomic group, infant characteristics (i.e., birth weight, gestational age, breastfeeding duration), carbohydrate intake (%), protein intake (%), monounsaturated fat intake (%), total energy intake (kcal); ^2^ adjusted for maternal characteristics (i.e., age at delivery, passive and active smoking during pregnancy, age at menarche, education, pre-pregnancy body mass index, parity), household highest socioeconomic group, infant characteristics (i.e., birth weight, gestational age, breastfeeding duration), carbohydrate intake (g), protein intake (g), monounsaturated fat intake (g), total energy intake (kcal).

**Table 3:** Associations of plasma phospholipid fatty acids at 7.5 years with puberty timing in the ALSPAC study ^1^.

Fatty Acids	Age at Genital/Breast Development		Age at Peak Height Velocity		Age at Voice Breaking/Menarche	
	Adjusted β Per SD (95% CI)	*p*	Adjusted β Per SD (95% CI)	*p*	Adjusted β Per SD (95% CI)	*p*
Boys	*n* = 2619		*n* = 2215		*n* = 3017	
n-6 polyunsaturated fatty acids						
Total	0.01 (−0.07, 0.09)	0.813	0.03 (−0.01, 0.07)	0.186	−0.01 (−0.09, 0.09)	0.965
Linoleic acid (18:2n6)	−0.01 (−0.08, 0.08)	0.957	0.03 (−0.01, 0.07)	0.158	0.01 (−0.09, 0.09)	0.990
γ-Linolenic acid (18:3n6)	0.05 (−0.02, 0.12)	0.175	−0.04 (−0.08, 0.01)	0.078	−0.02 (−0.10, 0.06)	0.593
Eicosadienoic acid (20:2n6)	−0.01 (−0.09, 0.07)	0.839	−0.01 (−0.05, 0.04)	0.935	0.01 (−0.08, 0.10)	0.886
Dihomo-γ-linolenic acid (20:3n6)	0.02 (−0.06, 0.09)	0.688	−0.03 (−0.07, 0.01)	0.189	0.02 (−0.07, 0.11)	0.710
Arachidonic acid (20:4n6)	0.02 (−0.05, 0.10)	0.521	0.02 (−0.02, 0.07)	0.299	−0.01 (−0.10, 0.07)	0.765
Docosatetraenoic acid (22:4n6)	0.04 (−0.03, 0.11)	0.243	0.03 (−0.01, 0.07)	0.193	0.05 (−0.04, 0.13)	0.279
Docosapentaenoic acid (22:5n6)	−0.03 (−0.10, 0.04)	0.347	0.03 (−0.01, 0.08)	0.110	0.04 (−0.04, 0.13)	0.301
Monounsaturated fatty acids						
Palmitoleic acid (16:1n7)	0.04 (−0.04, 0.12)	0.383	−0.05 (−0.10, −0.01)	0.020	−0.01 (−0.09, 0.08)	0.896
Oleic acid (18:1n9)	−0.03 (−0.11, 0.05)	0.492	0.01 (−0.03, 0.06)	0.584	0.04 (−0.05, 0.13)	0.374
Girls	*n* = 3204		*n* = 2509		*n* = 3414	
n-6 polyunsaturated fatty acids						
Total	−0.01 (−0.07, 0.07)	0.936	0.01 (−0.04, 0.04)	0.913	0.01 (−0.04, 0.05)	0.805
Linoleic acid (18:2n6)	0.02 (−0.05, 0.09)	0.656	0.01 (−0.03, 0.05)	0.640	0.02 (−0.03, 0.06)	0.498
γ-Linolenic acid (18:3n6)	0.01 (−0.07, 0.08)	0.893	−0.02 (−0.06, 0.02)	0.374	−0.03 (−0.09, 0.02)	0.199
Eicosadienoic acid (20:2n6)	−0.06 (−0.13, 0.01)	0.091	−0.03 (−0.07, 0.01)	0.125	−0.01 (−0.06, 0.04)	0.658
Dihomo-γ-linolenic acid (20:3n6)	−0.09 (−0.16, −0.02)	0.011	−0.07 (−0.11, −0.03)	6.3 × 10^−4^	−0.06 (−0.11, −0.01)	0.018
Arachidonic acid (20:4n6)	−0.02 (−0.10, 0.05)	0.501	0.01 (−0.04, 0.04)	0.861	−0.01 (−0.05, 0.04)	0.863
Docosatetraenoic acid (22:4n6)	0.03 (−0.04, 0.09)	0.408	0.02 (−0.02, 0.06)	0.240	0.02 (−0.03, 0.07)	0.367
Docosapentaenoic acid (22:5n6)	0.03 (−0.04, 0.09)	0.448	0.02 (−0.02, 0.06)	0.294	0.03 (−0.02, 0.08)	0.252
Monounsaturated fatty acids						
Palmitoleic acid (16:1n7)	−0.13 (−0.20, −0.06)	1.1 × 10^−4^	−0.06 (−0.10, −0.02)	0.001	−0.06 (−0.11, −0.01)	0.017
Oleic acid (18:1n9)	0.01 (−0.06, 0.08)	0.833	0.01 (−0.03, 0.05)	0.645	0.02 (−0.03, 0.06)	0.462

^1^ adjusted for maternal characteristics (i.e., age at delivery, passive and active smoking during pregnancy, age at menarche, education, pre-pregnancy body mass index, parity), household highest socioeconomic group, infant characteristics (i.e., birth weight, gestational age, breastfeeding duration), total energy intake at 6 years (kcal).

## Data Availability

The authors do not have the authority to share the data that support the findings of this study, due to ALSPAC data access permissions, but any researcher can apply to use ALSPAC data, including the variables used in this investigation. Information about access to ALSPAC data is given on their website: (http://www.bristol.ac.uk/alspac/researchers/access/) (accessed on 1 April 2021).

## Supplementary Materials

The following are available online at https://www.mdpi.com/article/10.3390/nu13061868/s1, Table S1. Comparisons of characteristics between excluded and included children in the ALSPAC study; Table S2. Correlations between dietary and plasma phospholipid polyunsaturated fatty acids in the ALSPAC study; Table S3. Correlations between plasma phospholipid polyunsaturated fatty acids and major monounsaturated fatty acids in the ALSPAC study; Table S4. Substitution of dietary monounsaturated fat for saturate fat intake predicted at 6 years old with puberty timing in the ALSPAC study; Table S5a. Associations of plasma fatty acids at 7.5 years with puberty timing, further adjusted for non-major food sources of polyunsaturated fatty acids in the ALSPAC study; Table S5b. Associations of plasma fatty acids at 7.5 years with puberty timing, additionally adjusted for food sources of polyunsaturated fatty acids in the ALSPAC study; Table S6. Associations of plasma fatty acids at 7.5 years with puberty timing, further adjusted for body mass index in the ALSPAC study; Table S7. Associations of self-reported dietary and objectively measured n-3 polyunsaturated fat with puberty timing in the ALSPAC study; Table S8. Associations of Vaccenic acid, saturated fatty acid categories and ratios of fatty acids at 7.5 years with puberty timing in the ALSPAC study; Table S9. Single nucleotide polymorphisms associated with Dihomo-γ-linolenic acid (20:3n6) and Palmitoleic acid (16:1n7) in the EPIC-InterAct study; Figure S1. Flowchart of the study inclusion in the ASLPAC cohort; Figure S2. Heatmaps that plot SNPs against specific fatty acids in the EPIC-InterAct study and low- and high -density lipoprotein in the UK Biobank study; Table S10. Associations of genetically predicted fatty acids with age at menarche.

## Appendix A

### Plausibility of Dietary Intakes

Dietary data from 3-day food diaries at each age for 223 to 329 individuals were excluded, owing to outlier values for total energy intakes on visual inspection of histograms (age 3: ≤349 kcal and ≥ 2617kcal; age 4: ≤514 kcal and ≥3263 kcal; age 7: ≤545 kcal and ≥3970 kcal) [34].

The rates of plausible reporters for 3-day food diaries based on:

the 95% CI [63] of the ratio of reported energy intake to estimated energy requirement [64] (0.79–1.21) [33,34] ranged from 69% to 76% at age 3 to 7.5 years in the 10% subcohorts (*n* = 772−863) [33,34] and at age 7.5 years in the whole cohort (*n* = 7017)

the calculated individual cut-off points [65] of basal metabolic rate estimated using sex-, age- and body weight-specific Schofield equations [66] was 79% at age 7.5 years in the whole cohort

## Appendix B

### Appendix B.1. Calculation of Puberty Timing Traits

Ages at onset of genital (G2) and breast (B2) development, and voice breaking were calculated from repeated annual self-reports between ages 8 to 17 years

If puberty was already present on the first report at age 8 years:

age at onset = age at 8 years report *minus* 6 months

If puberty was first reported after age 8 years:

age at onset = age at 8 years report *minus* 6 months *

* [the population average interval between the first report and the previous annual report]/2

If puberty was first reported after age 8 years but the one previous annual report was missing:

age at onset = age at first reported puberty *minus* 12 months **

** the population average interval between the first report and the previous annual report.

If puberty was first reported after age 8 years but the two previous annual reports were missing:

age at onset = age at first reported puberty *minus* 18 months ***

*** the population average interval between the first report and the previous annual report + 0.5x the interval between average ages at each annual report for each additional missing report.

If puberty was never reported (for voice breaking):

If puberty was not reported as attained at the last report at age 17 years: age at onset = age at 17 years report *plus* 6 months.

If puberty was not reported as attained and subsequent annual reports were missing: the aforementioned time intervals were added to the age at the last reported prepubertal status.

### Appendix B.2. Plausibility of Puberty Timing

Estimates of puberty timing (G2, B2, voice breaking) were performed in individuals whose reported measures followed a consistent sequential order with increasing age (52.2–69.0% of all included individuals).

Age at voice breaking was calculated in a further 11.8% of boys whose reported measures followed a consistent sequential order for at least three consecutive visits before or after an inconsistent report (e.g., ‘no voice breaking’ occurring after ‘partial or total voice breaking’).

For each of G2, B2, and voice breaking, timing of puberty could not be confidently calculated in 20.7–31.7% of individuals, due to inconsistent reports (i.e., non-sequential ordering of pubertal status and/or missing data).

Values for age at voice breaking (*n* = 27) or menarche (*n* = 39) were further excluded if these occurred earlier than age at G2 and B2, respectively.

## Appendix C

The European Prospective Investigation into Cancer and Nutrition Study (EPIC)-InterAct study [47] is a nested case-cohort study within eight of the participating countries of the EPIC cohort study: France, Italy, Spain, UK, the Netherlands, Germany, Sweden, and Denmark. The total sample of EPIC-InterAct included 12403 type-2 diabetes cases and selected a random subcohort of 16,835 individuals with baseline plasma samples, among 340,234 persons with 3.99 million person-years of follow-up (1991–2007) in the eight countries of the EPIC cohort. After exclusions due to prevalent diabetes (*n* = 548) and uncertain diabetes status (*n* = 133), the subcohort retained 16,154 individuals, of whom, 778 were the overlap group between the case-group and the subcohort as they had incident type 2 diabetes during follow-up. Of the 27,779 participants, 483 were further excluded due to missing data on fatty acid, leaving 27,286 adults. All participants gave written informed consent, and the study was approved by the local ethics committees in the participating countries and the Internal Review Board of the International Agency for Research on Cancer.

Plasma phospholipid fatty acids were profiled through solid phase extraction, hydrolysis, and methylation to convert phospholipid fatty acids into more volatile fatty acid methyl esters which were subsequently separated by gas chromatography (J&W HP-88, 30 m length, 0.25 mm internal diameter [Agilent Technologies, CA, USA]) equipped with flame ionization detection (7890N GC [Agilent Technologies]). Details on assay methods have been previously described [57,67,68].
