# Supplementary material for: Prepubertal Dietary and Plasma Phospholipid Fatty Acids Related to Puberty Timing: Longitudinal Cohort and Mendelian Randomization Analyses
Source: Nutrients. 2021 May 30;13(6):1868. doi: 10.3390/nu13061868 (PMC8228200; doi:10.3390/nu13061868)
Supplement: Supplementary file 1 [file nutrients-13-01868-s001.zip › nutrients-1209143-supplementary.pdf]

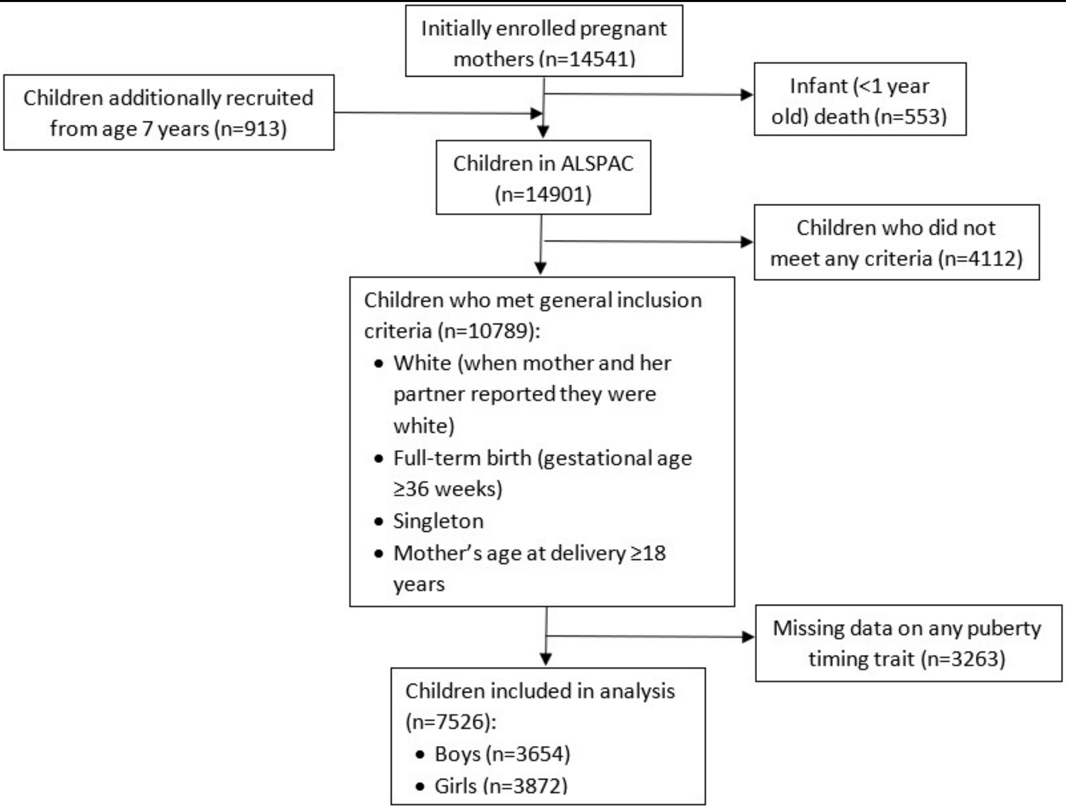

Figure S1. Flowchart of the study inclusion in the ASLPAC cohort.

Table S1. Comparisons of characteristics between excluded and included children in the ALSPAC study.

	Excluded (n=3263)	Included (n=7526)	*P*
**Maternal characteristics**			
Parity (n, %)			<0.001
0	1194 (39.1)	3332 (45.4)	
1	1126 (36.9)	2684 (36.6)	
2	498 (16.3)	981 (13.4)	
≥3	235 (7.7)	338 (4.6)	
Highest education level (n, %)			<0.001
None/CSE	930 (28.8)	1048 (14.0)	
Vocational	384 (11.9)	658 (8.8)	
O level	1112 (34.4)	2706 (36.0)	
A level	560 (17.3)	1917 (25.5)	
Degree	244 (7.6)	1181 (15.7)	
Active smoking during pregnancy (n, %)			<0.001
No	2187 (68.3)	6120 (82.4)	
Yes	1016 (31.7)	1309 (17.6)	
Passive smoking during pregnancy (n, %)			<0.001
None	874 (31.1)	2734 (43.3)	
<1 hour per day	226 (8.0)	576 (9.1)	
≥1 hour per day	1713 (60.9)	3011 (47.6)	
Highest household socioeconomic group at 18 weeks of gestation (n, %)			<0.001
Partly skilled and unskilled	350 (12.2)	491 (6.8)	
Skilled manual and non-manual	1934 (67.4)	4702 (65.2)	
Professional, managerial and technical	584 (20.4)	2019 (28.0)	
Age at delivery, years	27.16±4.87[1]	29.04±4.48	<0.001
Age at menarche, years	12.81±1.56	12.86±1.51	0.137
Pre-pregnancy body mass index, kg/m^2	23.08±4.08	22.97±3.75	0.231
**Child characteristics**			
Sex (n, %)			<0.001

Boys	1873 (57.4)	3654 (48.6)	
Girls	1390 (42.6)	3872 (51.5)	
Breastfeeding duration (n, %)			<0.001
Never	959 (35.3)	1542 (20.9)	
<3 months	884 (32.5)	2311 (31.4)	
3-<6 months	278 (10.2)	1036 (14.1)	
≥6 months	597 (22.0)	2478 (33.6)	
Gestational age, weeks	39.68±1.41	39.71±1.38	0.186
Birth weight, g	3471±493	3480±474	0.424
Dietary intakes predicted at age 6 years			
Total energy intake, kcal	1635±159	1616±158	<0.001
Total carbohydrate intake, g	204.6±21.2	201.9±20.7	<0.001
Total protein intake, g	56.0±5.3	55.6±5.6	0.008
Total fat intake, g	66.7±6.9	65.9±7.3	<0.001
Polyunsaturated fatty acids, g	10.9±1.1	10.8±1.2	0.005
Monounsaturated fatty acids, g	21.6±2.3	21.3±2.4	<0.001
Saturated fatty acids, g	27.1±3.7	26.7±3.8	<0.001
Plasma phospholipid fatty acids at 7.5 years			
Total fatty acids, μg/ml	2319.6±522.9	2328.6±536.9	0.736
Saturated fatty acids, μg%	29.2±3.3	29.2±3.2	0.917
Monounsaturated fatty acids, μg%	27.2±3.0	27.0±3.1	0.064
Polyunsaturated fatty acids, μg%	43.5±4.3	43.8±4.2	0.191

[1]Values are means ± SDs. CSE, Certificate of Secondary Education

Table S2. Correlations between dietary and plasma phospholipid polyunsaturated fatty acids in the ALSPAC study

Dietary intake at 6 years / Biomarkers at 7.5 years.	**Polyunsaturated fat (g)**		n-3 polyunsaturated fat (g)	
	**Boys**	Girls	Boys	Girls
n-6 polyunsaturated fatty acids (μg% of total fatty acids)	0.19	0.16	0.02	0.03
Linoleic acid (18:2n6)	0.22	0.20	0.03	0.04
γ-Linolenic acid (18:3n6)	0.01	-0.01	-0.04	-0.03
Eicosadienoic acid (20:2n6)	0.11	0.11	0.03	0.04
Dihomo-γ-linolenic acid (20:3n6)	0.01	-0.01	-0.03	-0.01
Arachidonic acid (20:4n6)	0.05	0.02	0.00	0.03
Docosatetraenoic acid (22:4n6)	0.03	0.00	-0.11	-0.10
Docosapentaenoic acid (22:5n6)	-0.02	-0.02	-0.12	-0.08
n-3 polyunsaturated fatty acids (μg% of total fatty acids)	-0.04	-0.05	0.22	0.25
α-Linolenic acid (18:3n3)	0.02	0.01	0.02	0.01
Eicosapentaenoic acid (20:5n3)	-0.11	-0.14	0.12	0.11
Docosapentaenoic acid (22:5n3)	-0.08	-0.08	-0.01	-0.01
Docosahexaenoic acid (22:6n3)	0.00	-0.01	0.28	0.32

Table S3. Correlations between plasma phospholipid polyunsaturated fatty acids and major monounsaturated fatty acids in the ALSPAC study

	n-6 PUFAs	18:2n6	18:3n6	20:2n6	20:3n6	20:4n6	22:4n6	22:5n6	n-3 PUFAs	18:3n3	20:5n3	22:5n3	22:6n3	MUFAs	16:1n7	18:1n9
n-6 PUFAs (µg% of total fatty acids)	1.00															
Linoleic acid (18:2n6)	0.92	1.00														
γ-Linolenic acid (18:3n6)	-0.04	-0.21	1.00													
Eicosadienoic acid (20:2n6)	0.40	0.29	0.09	1.00												
Dihomo-γ-linolenic acid (20:3n6)	0.38	0.17	0.34	0.53	1.00											
Arachidonic acid (20:4n6)	0.63	0.29	0.16	0.27	0.36	1.00										
Docosatetraenoic acid (22:4n6)	0.45	0.17	0.27	0.45	0.51	0.69	1.00									
Docosapentaenoic acid (22:5n6)	0.40	0.13	0.21	0.37	0.55	0.61	0.74	1.00								
n-3 PUFAs (µg% of total fatty acids)	0.24	0.06	0.04	0.28	0.23	0.49	0.23	0.19	1.00							
α-Linolenic acid (18:3n3)	-0.25	-0.16	-0.12	0.02	-0.21	-0.26	-0.28	-0.24	0.32	1.00						
Eicosapentaenoic acid (20:5n3)	0.04	-0.13	0.24	0.09	0.22	0.34	0.14	0.05	0.76	0.10	1.00					
Docosapentaenoic acid (22:5n3)	0.33	0.09	0.18	0.36	0.44	0.59	0.59	0.40	0.68	-0.03	0.62	1.00				
Docosahexaenoic acid (22:6n3)	0.40	0.21	-0.02	0.28	0.26	0.59	0.29	0.29	0.86	-0.09	0.53	0.52	1.00			
MUFAs (µg% of total fatty acids)	-0.68	-0.64	0.01	-0.12	-0.16	-0.44	-0.23	-0.15	-0.09	0.44	-0.06	-0.14	-0.31	1.00		
Palmitoleic acid (16:1n7)	-0.60	-0.60	0.26	-0.20	-0.08	-0.35	-0.18	-0.14	-0.23	-0.07	0.01	-0.16	-0.27	0.27	1.00	
Oleic acid (18:1n9)	-0.64	-0.58	-0.01	-0.12	-0.17	-0.46	-0.25	-0.16	-0.12	0.44	-0.11	-0.18	-0.33	0.99	0.16	1.00

PUFAs, polyunsaturated fatty acids; MUFA, monounsaturated fatty acids

Table S4. Substitution of dietary monounsaturated fat for saturate fat intake predicted at 6 years old with puberty timing in the ALSPAC study

Macronutrients	Age at genital/ breast development		Age at peak height velocity		Age at voice breaking/ menarche	
	Adjusted β (95% CI)	*P*	Adjusted β (95% CI)	*P*	Adjusted β (95% CI)	*P*
**Boys**	n=2619		n=2215		n=3017	
Nutrient density model (per 5% increase)[a]	-0.32 (-1.30, 0.66)	0.527	0.14 (-0.44, 0.72)	0.643	0.08 (-0.98, 1.14)	0.881
Residual model (per 10g increase)[b]	-0.53 (-1.57, 0.50)	0.313	0.03 (-0.51, 0.24)	0.933	0.07 (-1.05, 1.19)	0.901
**Girls**	n=3204		n=2509		n=3414	
Nutrient density model (per 5% increase)[a]	-0.66 (-1.58, 0.25)	0.154	-0.19 (-0.72, 0.33)	0.471	-0.21 (-0.87, 0.45)	0.534
Residual model (per 10g increase)[b]	-0.67 (-1.63, 0.30)	0.175	-0.19 (-0.74, 0.36)	0.498	-0.16 (-0.85, 0.53)	0.648

[a]Adjusted for maternal characteristics (i.e. age at delivery, passive and active smoking during pregnancy, age at menarche, education, pre-pregnancy body mass index, parity), household highest socioeconomic group, infant characteristics (i.e. birth weight, gestational age, breastfeeding duration), carbohydrate intake (%), protein intake (%), monounsaturated fat intake (%), total energy intake (kcal); [b]Adjusted for maternal characteristics (i.e. age at delivery, passive and active smoking during pregnancy, age at menarche, education, pre-pregnancy body mass index, parity), household highest socioeconomic group, infant characteristics (i.e. birth weight, gestational age, breastfeeding duration), carbohydrate intake (g), protein intake (g), monounsaturated fat intake (g), total energy intake (kcal)

Table S5a. Associations of plasma fatty acids at 7.5 years with puberty timing, further adjusted for non-major food sources of polyunsaturated fatty acids in the ALSPAC study

Fatty acids	Age at genital/ breast development		Age at peak height velocity		Age at voice breaking/ menarche	
	Adjusted β per SD (95% CI)	*P*	Adjusted β per SD (95% CI)	*P*	Adjusted β per SD (95% CI)	*P*
**Boys**	n=2619		n=2215		n=3017	
n-6 polyunsaturated fatty acids						
Total	0.01 (-0.07, 0.09)	0.868	0.03 (-0.01, 0.07)	0.206	0.01 (-0.08, 0.10)	0.900
Linoleic acid (18:2n6)	-0.01 (-0.09, 0.08)	0.904	0.03 (-0.01, 0.07)	0.176	0.01 (-0.08, 0.10)	0.811
γ-Linolenic acid (18:3n6)	0.05 (-0.02, 0.13)	0.168	-0.04 (-0.08, 0.01)	0.083	-0.03 (-0.11, 0.05)	0.510
Eicosadienoic acid (20:2n6)	-0.01 (-0.09, 0.07)	0.823	-0.01 (-0.05, 0.04)	0.949	0.01 (-0.08, 0.10)	0.822
Dihomo-γ-linolenic acid (20:3n6)	0.02 (-0.06, 0.09)	0.667	-0.03 (-0.07, 0.02)	0.211	0.01 (-0.08, 0.10)	0.790
Arachidonic acid (20:4n6)	0.02 (-0.05, 0.09)	0.559	0.02 (-0.02, 0.07)	0.324	-0.01 (-0.10, 0.07)	0.780
Docosatetraenoic acid (22:4n6)	0.04 (-0.03, 0.12)	0.242	0.03 (-0.01, 0.07)	0.201	0.04 (-0.04, 0.13)	0.328
Docosapentaenoic acid (22:5n6)	-0.03 (-0.10, 0.04)	0.345	0.04 (-0.01, 0.08)	0.108	0.04 (-0.04, 0.13)	0.307
Monounsaturated fatty acid						
Palmitoleic acid (16:1n7)	0.04 (-0.04, 0.12)	0.354	-0.05 (-0.10, -0.01)	0.025	-0.01 (-0.10, 0.08)	0.748
Oleic acid (18:1n9)	-0.02 (-0.11, 0.06)	0.551	0.01 (-0.03, 0.06)	0.553	0.03 (-0.06, 0.12)	0.460
**Girls**	n=3204		n=2509		n=3414	
n-6 polyunsaturated fatty acids						
Total	-0.01 (-0.08, 0.06)	0.726	-0.01 (-0.04, 0.4)	0.925	0.01 (-0.05, 0.05)	0.960
Linoleic acid (18:2n6)	0.01 (-0.07, 0.07)	0.947	0.01 (-0.04, 0.04)	0.821	0.01 (-0.04, 0.06)	0.660
γ-Linolenic acid (18:3n6)	0.01 (-0.06, 0.09)	0.765	-0.02 (-0.06, 0.02)	0.388	-0.04 (-0.09, 0.02)	0.196
Eicosadienoic acid (20:2n6)	-0.06 (-0.13, 0.01)	0.090	-0.03 (-0.07, 0.01)	0.121	-0.01 (-0.06, 0.03)	0.654
Dihomo-γ-linolenic acid (20:3n6)	-0.08 (-0.15, -0.01)	0.020	-0.07 (-0.11, -0.03)	5.7E-4	-0.06 (-0.11, -0.01)	0.014
Arachidonic acid (20:4n6)	-0.02 (-0.09, 0.05)	0.519	0.01 (-0.04, 0.04)	0.886	-0.01 (-0.05, 0.05)	0.875
Docosatetraenoic acid (22:4n6)	0.03 (-0.03, 0.10)	0.331	0.02 (-0.02, 0.06)	0.244	0.02 (-0.03, 0.07)	0.377
Docosapentaenoic acid (22:5n6)	0.03 (-0.04, 0.10)	0.373	0.02 (-0.02, 0.06)	0.298	0.03 (-0.02, 0.08)	0.290
Monounsaturated fatty acid						
Palmitoleic acid (16:1n7)	-0.12 (-0.19, -0.06)	3.4E-4	-0.06 (-0.10, -0.02)	0.002	-0.06 (-0.11, -0.01)	0.020
Oleic acid (18:1n9)	0.02 (-0.05, 0.09)	0.642	0.02 (-0.02, 0.06)	0.458	0.02 (-0.02, 0.07)	0.306

Adjusted for maternal characteristics (i.e. age at delivery, passive and active smoking during pregnancy, age at menarche, education, pre-pregnancy body mass index, parity), household highest socioeconomic group, infant characteristics (i.e. birth weight, gestational age, breastfeeding duration), total energy intake at 6 years (kcal), *red meats (g), chicken (g), fruits and vegetables (g), dairy and eggs (g) and sugar confectionary (g)*

Table S5b. Associations of plasma fatty acids at 7.5 years with puberty timing, additionally adjusted for food sources of polyunsaturated fatty acids in the ALSPAC study

Fatty acids	Age at genital/ breast development		Age at peak height velocity		Age at voice breaking/ menarche	
	Adjusted β per SD (95% CI)	*P*	Adjusted β per SD (95% CI)	*P*	Adjusted β per SD (95% CI)	*P*
**Boys**	n=2619		n=2215		n=3017	
n-6 polyunsaturated fatty acids						
Total	0.01 (-0.07, 0.09)	0.867	0.03 (-0.01, 0.07)	0.202	0.01 (-0.08, 0.10)	0.889
Linoleic acid (18:2n6)	-0.01 (-0.08, 0.08)	0.932	0.03 (-0.01, 0.07)	0.153	0.01 (-0.08, 0.10)	0.800
γ-Linolenic acid (18:3n6)	0.05 (-0.02, 0.12)	0.185	-0.04 (-0.08, 0.01)	0.064	-0.03 (-0.11, 0.05)	0.493
Eicosadienoic acid (20:2n6)	-0.01 (-0.09, 0.07)	0.831	-0.01 (-0.05, 0.04)	0.942	0.01 (-0.08, 0.10)	0.778
Dihomo-γ-linolenic acid (20:3n6)	0.01 (-0.06, 0.09)	0.715	-0.03 (-0.07, 0.01)	0.167	0.01 (-0.08, 0.10)	0.799
Arachidonic acid (20:4n6)	0.02 (-0.05, 0.09)	0.594	0.02 (-0.02, 0.06)	0.348	-0.01 (-0.10, 0.08)	0.795
Docosatetraenoic acid (22:4n6)	0.04 (-0.04, 0.11)	0.342	0.02 (-0.02, 0.06)	0.325	0.04 (-0.05, 0.13)	0.355
Docosapentaenoic acid (22:5n6)	-0.04 (-0.11, 0.03)	0.217	0.03 (-0.02, 0.07)	0.199	0.04 (-0.04, 0.13)	0.333
Monounsaturated fatty acid						
Palmitoleic acid (16:1n7)	0.04 (-0.05, 0.12)	0.392	-0.06 (-0.10, -0.01)	0.018	-0.02 (-0.11, 0.07)	0.730
Oleic acid (18:1n9)	-0.03 (-0.11, 0.05)	0.501	0.01 (-0.04, 0.05)	0.757	0.03 (-0.06, 0.12)	0.493
**Girls**	n=3204		n=2509		n=3414	
n-6 polyunsaturated fatty acids						
Total	0.01 (-0.08, 0.06)	0.745	-0.01 (-0.04, 0.04)	0.958	0.01 (-0.05, 0.05)	0.947
Linoleic acid (18:2n6)	0.01 (-0.07, 0.07)	0.920	0.01 (-0.03, 0.05)	0.790	0.01 (-0.04, 0.06)	0.655
γ-Linolenic acid (18:3n6)	0.01 (-0.06, 0.08)	0.788	-0.02 (-0.06, 0.02)	0.371	-0.04 (-0.09, 0.02)	0.197
Eicosadienoic acid (20:2n6)	-0.06 (-0.13, 0.01)	0.100	-0.03 (-0.06, 0.01)	0.134	-0.01 (-0.05, 0.04)	0.708
Dihomo-γ-linolenic acid (20:3n6)	-0.08 (-0.15, -0.01)	0.018	-0.07 (-0.11, -0.03)	5.2E-4	-0.06 (-0.11, -0.01)	0.015
Arachidonic acid (20:4n6)	-0.02 (-0.09, 0.05)	0.528	0.01 (-0.04, 0.04)	0.857	-0.01 (-0.05, 0.05)	0.895
Docosatetraenoic acid (22:4n6)	0.03 (-0.04, 0.09)	0.391	0.02 (-0.02, 0.06)	0.289	0.02 (-0.03, 0.07)	0.376
Docosapentaenoic acid (22:5n6)	0.03 (-0.04, 0.10)	0.431	0.02 (-0.02, 0.06)	0.338	0.03 (-0.02, 0.08)	0.289
Monounsaturated fatty acid						
Palmitoleic acid (16:1n7)	-0.12 (-0.19, -0.06)	2.8E-4	-0.06 (-0.10, -0.02)	0.002	-0.06 (-0.11, -0.01)	0.020
Oleic acid (18:1n9)	0.01 (-0.06, 0.08)	0.713	-0.03 (-0.07, 0.01)	0.161	0.02 (-0.02, 0.07)	0.329

Adjusted for maternal characteristics (i.e. age at delivery, passive and active smoking during pregnancy, age at menarche, education, pre-pregnancy body mass index, parity), household highest socioeconomic group, infant characteristics (i.e. birth weight, gestational age, breastfeeding duration), total energy intake at 6 years (kcal), *red meats (g), chicken (g), fruits and vegetables (g), dairy and eggs (g) and sugar confectionary (g), fish (g) and cereals and nuts (g)*

Table S6. Associations of plasma fatty acids at 7.5 years with puberty timing, further adjusted for body mass index in the ALSPAC study

Fatty acids	Age at genital/ breast development		Age at peak height velocity		Age at voice breaking/ menarche	
	Adjusted β per SD (95% CI)	*P*	Adjusted β per SD (95% CI)	*P*	Adjusted β per SD (95% CI)	*P*
**Boys**	n=2619		n=2215		n=3017	
n-6 polyunsaturated fatty acids						
Total	0.02 (-0.06, 0.10)	0.676	0.02 (-0.03, 0.06)	0.452	-0.01 (-0.10, 0.08)	0.793
Linoleic acid (18:2n6)	0.01 (-0.07, 0.09)	0.848	0.01 (-0.03, 0.05)	0.615	-0.01 (-0.10, 0.07)	0.822
γ-Linolenic acid (18:3n6)	0.04 (-0.03, 0.12)	0.235	-0.02 (-0.06, 0.02)	0.391	-0.01 (-0.09, 0.07)	0.837
Eicosadienoic acid (20:2n6)	-0.01 (-0.09, 0.07)	0.782	0.01 (-0.04, 0.05)	0.861	0.01 (-0.08, 0.10)	0.819
Dihomo-γ-linolenic acid (20:3n6)	0.01 (-0.07, 0.08)	0.867	-0.01 (-0.05, 0.03)	0.676	0.03 (-0.06, 0.12)	0.473
Arachidonic acid (20:4n6)	0.02 (-0.05, 0.10)	0.513	0.02 (-0.02, 0.07)	0.248	-0.01 (-0.10, 0.07)	0.782
Docosatetraenoic acid (22:4n6)	0.04 (-0.03, 0.12)	0.227	0.03 (-0.02, 0.07)	0.216	0.04 (-0.04, 0.13)	0.291
Docosapentaenoic acid (22:5n6)	-0.03 (-0.10, 0.04)	0.448	0.02 (-0.02, 0.06)	0.285	0.03 (-0.05, 0.12)	0.420
Monounsaturated fatty acid						
Palmitoleic acid (16:1n7)	0.02 (-0.06, 0.10)	0.640	-0.02 (-0.06, 0.03)	0.432	0.02 (-0.07, 0.11)	0.630
Oleic acid (18:1n9)	-0.03 (-0.11, 0.05)	0.429	0.02 (-0.03, 0.06)	0.445	0.05 (-0.04, 0.13)	0.314
**Girls**	n=3204		n=2509		n=3414	
n-6 polyunsaturated fatty acids						
Total	-0.43 (-1.04, 0.17)	0.163	-0.02 (-0.06, 0.01)	0.237	-0.02 (-0.07, 0.03)	0.408
Linoleic acid (18:2n6)	-0.05 (-0.11, 0.02)	0.155	-0.02 (-0.06, 0.01)	0.211	-0.02 (-0.06, 0.03)	0.492
γ-Linolenic acid (18:3n6)	0.04 (-0.03, 0.10)	0.278	-0.01 (-0.04, 0.04)	0.943	-0.02 (-0.07, 0.03)	0.534
Eicosadienoic acid (20:2n6)	-0.04 (-0.11, 0.02)	0.226	-0.02 (-0.05, 0.01)	0.241	-0.01 (-0.05, 0.04)	0.939
Dihomo-γ-linolenic acid (20:3n6)	-0.05 (-0.12, 0.01)	0.096	-0.05 (-0.09, -0.01)	0.008	-0.04 (-0.09, 0.01)	0.089
Arachidonic acid (20:4n6)	-0.03 (-0.09, 0.04)	0.423	0.01 (-0.03, 0.04)	0.846	-0.01 (-0.05, 0.04)	0.793
Docosatetraenoic acid (22:4n6)	-0.01 (-0.06, 0.06)	0.963	0.01 (-0.03, 0.05)	0.625	0.01 (-0.04, 0.06)	0.754
Docosapentaenoic acid (22:5n6)	-0.02 (-0.09, 0.04)	0.513	-0.01 (-0.04, 0.04)	0.937	0.01 (-0.04, 0.04)	0.907
Monounsaturated fatty acid						
Palmitoleic acid (16:1n7)	-0.02 (-0.08, 0.04)	0.564	-0.01 (-0.04, 0.03)	0.780	-0.01 (-0.05, 0.05)	0.988
Oleic acid (18:1n9)	0.04 (-0.03, 0.10)	0.246	0.02 (-0.01, 0.06)	0.184	0.03 (-0.01, 0.08)	0.163

Adjusted for maternal characteristics (i.e. age at delivery, passive and active smoking during pregnancy, age at menarche, education, pre-pregnancy body mass index, parity), household highest socioeconomic group, infant characteristics (i.e. birth weight, gestational age, breastfeeding duration), total energy intake at 6 years (kcal), *body mass index at 7.5 years (kg/m²)*

Table S7. Associations of self-reported dietary and objectively measured n-3 polyunsaturated fat with puberty timing in the ALSPAC study

	Age at genital/ breast development		Age at peak height velocity		Age at voice breaking/ menarche	
	Adjusted β per SD (95% CI)	*P*	Adjusted β per SD (95% CI)	*P*	Adjusted β per SD (95% CI)	*P*
Dietary intake at 6 years						
Boys	n=2619		n=2215		n=3017	
n-3 polyunsaturated fat	-0.07 (-0.14, -0.01)	0.042	-0.02 (-0.06, 0.02)	0.236	-0.07 (-0.14, 0.01)	0.062
Eicosapentaenoic acid	-0.07 (-0.14, -0.01)	0.038	-0.02 (-0.06, 0.02)	0.257	-0.08 (-0.15, -0.01)	0.039
Docosahexaenoic acid	-0.07 (-0.14, -0.01)	0.033	-0.02 (-0.06, 0.02)	0.270	-0.07 (-0.14, 0.01)	0.064
Girls	n=3204		n=2509		n=3414	
n-3 polyunsaturated fat	-0.04 (-0.10, 0.01)	0.100	-0.01 (-0.04, 0.03)	0.721	0.01 (-0.02, 0.05)	0.472
Eicosapentaenoic acid	-0.04 (-0.09, 0.02)	0.180	0.01 (-0.03, 0.03)	0.945	0.02 (-0.02, 0.06)	0.304
Docosahexaenoic acid	-0.04 (-0.10, 0.01)	0.105	-0.01 (-0.04, 0.02)	0.692	0.01 (-0.02, 0.05)	0.489
Plasma fatty acids at 7.5 years						
Boys						
n-3 polyunsaturated fatty acids	0.01 (-0.06, 0.08)	0.793	-0.03 (-0.08, 0.01)	0.111	-0.04 (-0.12, 0.05)	0.411
Eicosapentaenoic acid (20:5n3)	0.02 (-0.05, 0.09)	0.608	-0.02 (-0.06, 0.02)	0.222	-0.08 (-0.16, 0.01)	0.061
Docosahexaenoic acid (22:6n3)	-0.01 (-0.08, 0.07)	0.873	-0.03 (-0.08, 0.01)	0.124	-0.04 (-0.12, 0.05)	0.416
Girls						
n-3 polyunsaturated fatty acids	-0.05 (-0.11, 0.02)	0.161	-0.01 (-0.04, 0.04)	0.936	-0.02 (-0.06, 0.03)	0.476
Eicosapentaenoic acid (20:5n3)	-0.06 (-0.13, 0.01)	0.089	-0.01 (-0.04, 0.04)	0.878	-0.03 (-0.07, 0.02)	0.282
Docosahexaenoic acid (22:6n3)	-0.04 (-0.10, 0.03)	0.256	-0.01 (-0.05, 0.03)	0.573	-0.02 (-0.07, 0.02)	0.371

[a]Adjusted for maternal characteristics (i.e. age at delivery, passive and active smoking during pregnancy, age at menarche, education, pre-pregnancy body mass index, parity), household highest socioeconomic group, infant characteristics (i.e. birth weight, gestational age, breastfeeding duration), total energy intake at 6 years (kcal)

Table S8. Associations of Vaccenic acid, saturated fatty acid categories and ratios of fatty acids at 7.5 years with puberty timing in the ALSPAC study

	Age at genital/ breast development		Age at peak height velocity		Age at voice breaking/ menarche	
	Adjusted β per SD (95% CI)	*P*	Adjusted β per SD (95% CI)	*P*	Adjusted β per SD (95% CI)	*P*
Boys	n=2619		n=2215		n=3017	
MUFA						
Vaccenic acid (18:1n7)	-0.01 (-0.09, 0.07)	0.754	0.01 (-0.04, 0.05)	0.780	0.03 (-0.05, 0.12)	0.446
Erucic acid (20:1n9)	-0.03 (-0.11, 0.05)	0.435	0.02 (-0.02, 0.06)	0.375	0.01 (-0.08, 0.09)	0.952
Nervonic acid (24:1n9)	0.04 (-0.04, 0.11)	0.337	-0.04 (-0.08, 0.01)	0.109	-0.05 (-0.13, 0.03)	0.240
SFA						
Short-even-chain (14:0, 16:0, 18:0)	0.01 (-0.08, 0.08)	0.968	-0.03 (-0.07, 0.01)	0.169	-0.02 (-0.11, 0.07)	0.645
Long-even-chain (20:0, 22:0, 24:0)	0.04 (-0.04, 0.11)	0.330	0.01 (-0.03, 0.05)	0.654	-0.03 (-0.11, 0.06)	0.532
Ratios of n-6 PUFA						
Ratio: 18:3n6 / 18:2n6 (Δ6 desaturase)	0.05 (-0.03, 0.12)	0.208	-0.04 (-0.08, -0.01)	0.041	-0.02 (-0.10, 0.06)	0.678
Ratio: 20:4n6 / 20:3n6 (Δ5 desaturase)	0.01 (-0.06, 0.09)	0.756	0.04 (-0.01, 0.09)	0.070	-0.03 (-0.11, 0.06)	0.546
Ratio: 20:3n6/ 18:2n6 (DGLA to LA)	0.01 (-0.06, 0.09)	0.707	-0.04 (-0.08, 0.01)	0.052	0.02 (-0.06, 0.11)	0.601
Stearoyl-CoA desaturase-1						
Ratio: 16:1n7 / 16:0	0.05 (-0.03, 0.12)	0.252	-0.06 (-0.11, -0.01)	0.011	0.01 (-0.09, 0.09)	0.990
Ratio: 18:1n9 / 18:0	-0.02 (-0.09, 0.06)	0.690	0.03 (-0.01, 0.08)	0.175	0.05 (-0.04, 0.14)	0.263
Ratio: total n-6 PUFAs / total n-3 PUFAs	-0.02 (-0.09, 0.05)	0.522	0.04 (0.01, 0.09)	0.032	0.02 (-0.06, 0.09)	0.704
Ratio: total MUFAs / total SFAs	-0.02 (-0.10, 0.06)	0.623	0.02 (-0.02, 0.07)	0.304	0.05 (-0.04, 0.13)	0.284
Girls	n=3204		n=2509		n=3414	
MUFA						
Vaccenic acid (18:1n7)	-0.07 (-0.14, 0.01)	0.064	-0.03 (-0.07, 0.01)	0.137	-0.01 (-0.06, 0.05)	0.793
Erucic acid (20:1n9)	0.03 (-0.04, 0.10)	0.341	0.02 (-0.02, 0.06)	0.382	0.02 (-0.03, 0.07)	0.436
Nervonic acid (24:1n9)	-0.04 (-0.10, 0.03)	0.298	-0.01 (-0.05, 0.03)	0.631	-0.03 (-0.08, 0.02)	0.251
SFA						
Short-even-chain (14:0, 16:0, 18:0)	0.03 (-0.04, 0.10)	0.399	-0.01 (-0.04, 0.04)	0.928	-0.01 (-0.06, 0.04)	0.754
Long-even-chain (20:0, 22:0, 24:0)	0.01 (-0.05, 0.08)	0.691	0.02 (-0.02, 0.06)	0.426	-0.01 (-0.06, 0.05)	0.861
Ratios of n-6 PUFA						
Ratio: 18:3n6 / 18:2n6 (Δ6 desaturase)	-0.01 (-0.08, 0.07)	0.901	-0.02 (-0.06, 0.02)	0.317	-0.04 (-0.09, 0.02)	0.192
Ratio: 20:4n6 / 20:3n6 (Δ5 desaturase)	0.06 (-0.01, 0.13)	0.068	0.06 (0.02, 0.10)	0.001	0.04 (-0.01, 0.09)	0.070
Ratio: 20:3n6/ 18:2n6 (DGLA to LA)	-0.09 (-0.16, -0.02)	0.001	-0.07 (-0.11, -0.03)	5.7E-4	-0.06 (-0.11, -0.01)	0.016
Stearoyl-CoA desaturase-1						
Ratio: 16:1n7 / 16:0	-0.18 (-0.24, -0.11)	7.7E-8	-0.08 (-0.11, -0.04)	4.2E-5	-0.07 (-0.12, -0.03)	0.003
Ratio: 18:1n9 / 18:0	-0.01 (-0.08, 0.05)	0.690	0.01 (-0.03, 0.04)	0.772	0.02 (-0.02, 0.07)	0.360
Ratio: total n-6 PUFAs / total n-3 PUFAs	0.05 (-0.01, 0.12)	0.118	0.01 (-0.03, 0.04)	0.827	0.02 (-0.03, 0.06)	0.455
Ratio: total MUFAs / total SFAs	-0.04 (-0.11, 0.03)	0.310	0.01 (-0.04, 0.04)	0.916	0.01 (-0.04, 0.06)	0.652

Adjusted for maternal characteristics (i.e. age at delivery, passive and active smoking during pregnancy, age at menarche, education, pre-pregnancy body mass index, parity), household highest socioeconomic group, infant characteristics (i.e. birth weight, gestational age, breastfeeding duration), total energy intake at 6 years (kcal)

MUFA, monounsaturated fatty acid; SFA, saturated fatty acid; PUFA, polyunsaturated fatty acids; DGLA, Dihomo-γ-linolenic acid; LA, Linoleic acid

Table S9. Single nucleotide polymorphisms associated with Dihomo-γ-linolenic acid (20:3n6) and Palmitoleic acid (16:1n7) in the EPIC-InterAct study

Chromosome	SNP	Position	Locus	Effect allele	Other allele	Effect	Standard error	P value	Frequency of effect allele
**Dihomo-γ-linolenic acid (20:3n6)**									
Primary instrument									
16	rs12928099	15150505	PCXDC1	A	C	0.30	0.01	2.3×10^{-196}	0.307
Secondary instruments									
8	rs721399	18259366	NAT2	T	C	0.06	0.01	1.3×10^{-8}	0.722
11	rs499974	75455021	DGAT2	A	C	-0.09	0.01	2.3×10^{-13}	0.176
Excluded due to pleiotropy									
11	rs968567	61595564	FADS1	T	C	0.50	0.01	7.2×10^{-308}	0.160
19	rs8107974	19388500	TM6SF2	A	T	0.10	0.02	1.5×10^{-8}	0.921
**Palmitoleic acid (16:1n7)**									
Primary instrument									
10	rs603424	102075479	SCD	A	G	-0.15	0.01	6.2×10^{-38}	0.212
Secondary instruments									
9	rs4962238	140358556	NSMF	T	C	0.11	0.02	3.3×10^{-8}	0.079
14	rs116915125	26984435	NOVA1	A	G	0.27	0.05	1.1×10^{-8}	0.015
Excluded due to pleiotropy									
2	rs1260326	27730940	GCKR	T	C	0.09	0.01	4.4×10^{-19}	0.406
4	rs1229984	100239319	ADH1A	T	C	-0.14	0.02	5.3×10^{-9}	0.046
10	rs2792736	113921159	GPAM	A	T	-0.06	0.01	4.9×10^{-8}	0.700
11	rs174566	61592362	FADS1	A	G	-0.11	0.01	1.4×10^{-29}	0.670

Figure S2. Heatmaps that plot SNPs against specific fatty acids in the EPIC-InterAct study and low- and high -density lipoprotein in the UK Biobank study

(a) Dihomo-γ-linolenic acid (20:3n6)

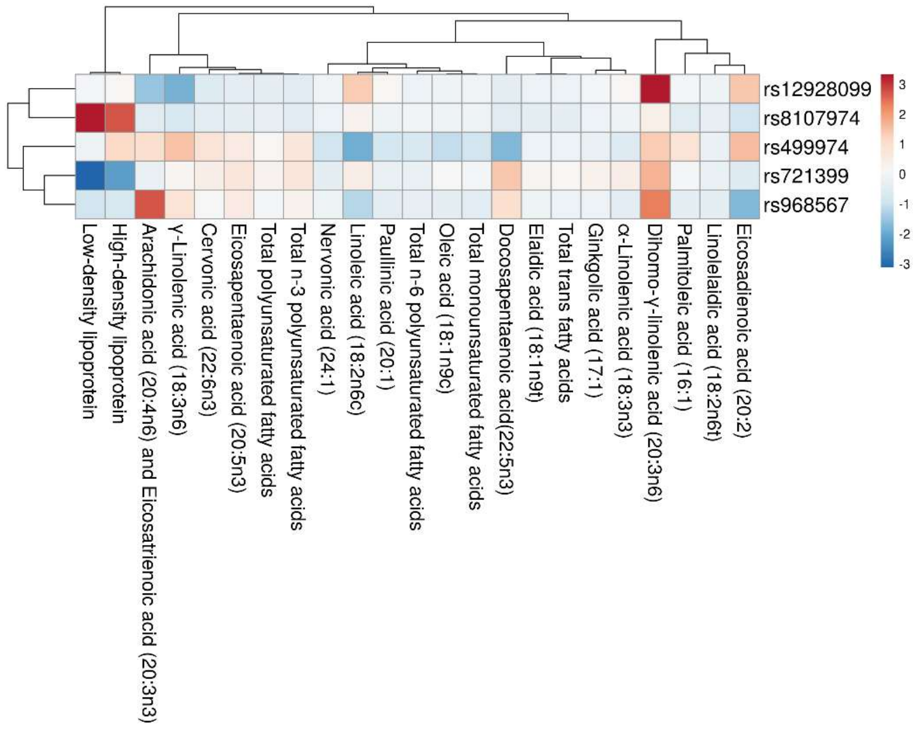

(b) Palmitoleic acid (16:1n7)

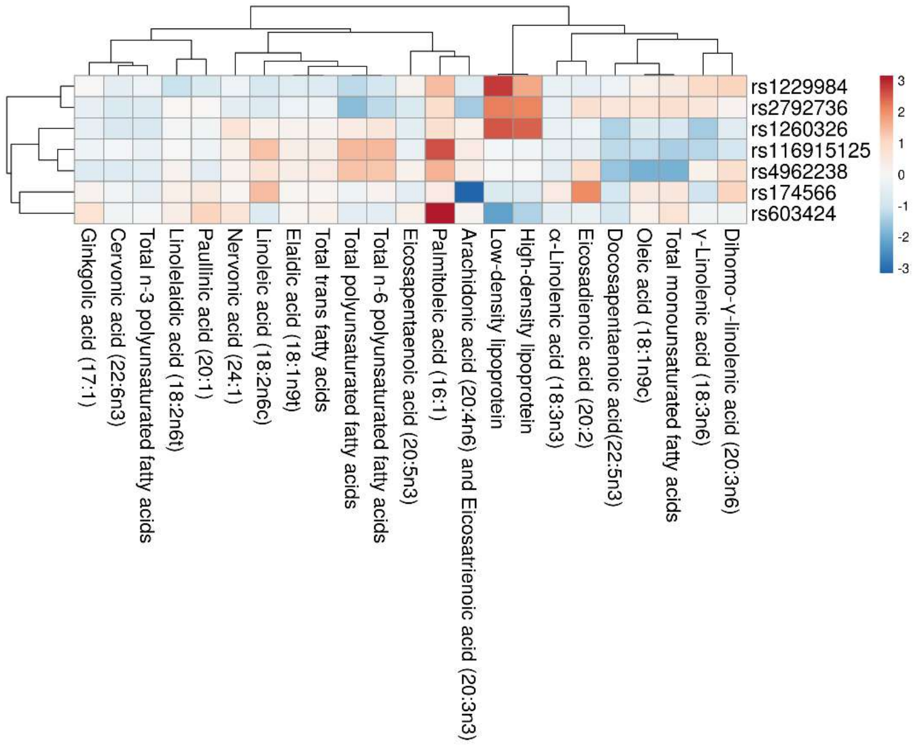

Table S10. Associations of genetically predicted fatty acids with age at menarche

Fatty acids	Number of SNP	β (95% CI)	*P*
**Dihomo-γ-linolenic acid (20:3n6)**			
rs12928099	1	-0.05 (-0.09, -0.01)	0.019
Inverse variance weighted	3	-0.04 (-0.09, 0.02)	0.166
Weighted Median	3	-0.05 (-0.09, -0.01)	0.020
Penalised weighted median	3	-0.04 (-0.09, -0.01)	0.019
**Palmitoleic acid (16:1n7)**			
rs603424	1	0.08 (-0.01, 0.16)	0.082
Inverse variance weighted	2	0.05 (-0.09, 0.20)	0.472
